# Anterior occlusion in shortened dental arches

**DOI:** 10.1007/s00784-021-04317-5

**Published:** 2021-12-10

**Authors:** Elżbieta Pacek, Michael H. Walter

**Affiliations:** Prosthodontics, Faculty of Medicine Carl Gustav Carus, Technische Universität Dresden, Fetscherstr. 74, 01307 Dresden, Germany

**Keywords:** Dental arch, Tooth loss, Dental occlusion, Bite force

## Abstract

**Objectives:**

The aim of this study was to examine the occlusion of anterior teeth in individuals with shortened dental arch (SDA).

**Material and methods:**

In a case–control clinical study, 41 individuals with SDA and 41 individuals with complete dental arch (CDA) participated. The CDA control group was matched for age and gender. Testing for occlusal contacts of anterior maxillary teeth was conducted by biting on foil strips (8 µm) with subjectively normal bite force (NBF) and maximal bite force (MBF). The data was analyzed on individual and tooth levels.

**Results:**

The median rates of anterior maxillary teeth with occlusal contacts were 0.67 (NBF) and 0.83 (MBF) in the SDA group and 0.50 (NBF) and 0.83 (MBF) in the CDA group. Within both groups, the contact rates were significantly higher in MBF. The group difference with NBF was significant. A generalized linear model showed that the odds of an anterior maxillary tooth to have an occlusal contact were greater in the SDA both for NBF with an odds ratio (OR) 2.277 and MBF with an OR 1.691.

**Conclusions:**

The findings suggest effective compensatory mechanisms relative to the occlusal function in individuals with SDA.

**Clinical relevance:**

The study delivers further evidence regarding the SDA concept as a viable option in the management of posterior tooth loss.

## Introduction

The shortened dental arch (SDA) concept has been comprehensively researched and widely supported. The available papers focus on occlusal stability, masticatory function, prevalence of temporomandibular joint disorders (TMDs), the effect of partial removable dental prostheses (PRDPs), and oral comfort [1, 2]. Four bilaterally symmetrical posterior occlusal units may have sufficient adaptive capacity to maintain oral function [3]. It has been stated that PRDPs show beneficial influence on oral health-related quality of life (OHQoL) among patients with SDA only if the anterior dentition is interrupted and needs restoration as well [4]. It was also assumed that placing PRDPs in patients with SDA could lead to overtreatment. In 725 Tanzanian adults with different variations of SDAs and 125 adults with a complete dental arch (CDA), four pairs of occluding premolars and at least one pair of molars were found to be enough to chew most food types [5]. The risk of TMDs caused by SDAs appears to be low [6, 7]. Patients with SDA did not report worse oral comfort [8]. No difference in OHQoL was reported in individuals with SDA and CDA [9].

In a long-term follow-up study, a considerable proportion of SDA patients upheld their status leading to the conclusion that maintaining the SDA is a durable concept which can last up to 27 years or more [10]. In a randomized clinical trial on the SDA, two treatment options were compared [11]. Missing molars and when missing also the second premolar were either replaced with a PRDP or the premolar occlusion with all premolars was preserved or restored with fixed dental prostheses (FDPs). Overall, there were only minor differences between the treatments concerning a variety of outcomes such as tooth loss, periodontal health, and OHQoL over 10 years [12–14]. The findings delivered further evidence in favor of the SDA concept.

The widely accepted current view of occlusion is based on a physiological occlusion which develops during individual growth and forms by inner and outer factors that not always correspond with perfection [15]. The loss of molar support could be seen as one of these factors. A study in China focused on tooth wear among individuals with SDA [16]. Premolars had a tendency to greater occlusal wear. An association between tooth wear in the anterior dentition and less support in the posterior area was found albeit not statistically significant. Occlusal changes after molar loss as interdental spacing and increasing numbers of occlusal contacts in the anterior dentition were described as self-limiting, having an adaptive character and an expression of a new equilibrium [17]. The adaptive changes regarding occlusion in SDAs have only been sporadically researched [16, 18, 19]. The aim of this study was to investigate the anterior occlusion in individuals with SDA. The study hypothesis was that individuals with SDA have more occlusal contacts in anterior teeth.

## Materials and methods

This study was designed as a case–control study with case and control group. All eligible individuals were notified of the goals and methods of the study as well as the risks and voluntary nature of participation. An informed consent form was signed prior to participation.

### Participants

#### SDA group

The case group included individuals at least 40 years of age with an SDA in one jaw to at least the first premolar with uninterrupted anterior dentition. In the opposing jaw, all anterior teeth had to be present. In the SDA, at least one premolar per side had to be in occlusal contact in maximal intercuspation. Missing teeth replaced by implant born restorations and FDPs were treated as natural teeth. The SDA had to be present for at least 3 years. Ongoing orthodontic treatment and PRDPs were exclusion criteria. Intermaxillary relationship, periodontal conditions, and tooth mobility were not taken into consideration.

#### CDA group

The control group included individuals matched by year of birth and gender with complete dental arches in both jaws to at least first molars. Implant born restorations and FDPs were treated as natural teeth.

### Recruitment

Participants with a dental status fulfilling the criteria for the SDA were extracted from the patient database of the dental clinic of the University Hospital Carl Gustav Carus at the Technische Universität Dresden. An electronic search was complemented by hand search and screening of patient files and radiographs to ensure eligibility. The potential participants were contacted by phone. The recruitment of the control group followed the same regimen.

### Variables

After recording the dental status, the distribution of occlusal contacts was examined. Shimstock foil (Hanel Shimstockfolie, 8 µm, 8 mm × 5 mm, Coltene Whaledent, Germany) was used for identifying occlusal contacts. The foil was cut into small pieces which fitted the areas relevant for occlusal contacts. Individuals were examined seated upright in a dental chair. They were asked to bite down on to the foil to reach maximal (habitual) intercuspation. The movement of the mandible remained unguided. An occlusal contact was counted when the foil was wedged between teeth and could not be removed with moderate pull of the examiner. If the foil slipped out, it was counted as no occlusal contact. The anterior contacts were always related to the maxillary teeth. The procedure was performed in two rounds, once with normal bite force (NBF), request to “bite normal,” and a second time with maximal bite force (MBF), request to “bite with full strength.”

### Statistics

Statistic analyses comprised descriptive statistics, tests of normality (Kolmogorov–Smirnov Test, Shapiro–Wilk Test), Wilcoxon Test, Mann–Whitney *U* test, and generalized linear models. Tooth contacts were analyzed on the tooth and individual level. An individual contact rate (CR) was determined by dividing the number of maxillary anterior teeth with contact by the total number of maxillary anterior teeth. The significance level was set at *P* = 0.05. The analyses were conducted using the software IBM SPSS Statistics (version 27, IBM Corporation). The sample size calculation based on a paired samples *T*-test. A difference of ∆CR = 0.4 was considered appropriate and clinically relevant. To reach significance for this difference, samples of 40 participants per group were calculated.

## Results

The SDA and CDA groups consisted of 41 individuals each. The groups included 42 female and 40 male participants aged 74.6 ± 8.2 years (mean ± SD). The youngest individual was 55 and the oldest 88 years old. The mean number of teeth was 22.5 in the SDA group and 27.7 in the CDA group.

### Tooth-related contacts

The individuals in the SDA group exhibited a total of 924 teeth. Under NBF, 591 (64%) teeth had occlusal contacts. Under MBF, 654 teeth (71%) had occlusal contacts. Very few molars had occlusal contacts because of having premolars in SDAs as antagonists. The individuals in the CDA group exhibited a total of 1134 teeth. Under NBF, 797 (70%) teeth had occlusal contacts. Under MBF, 938 teeth (82%) had occlusal contacts.

**Fig. 1:**
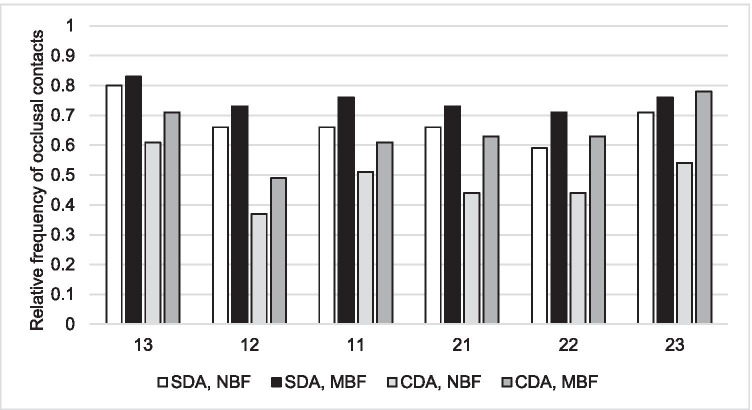
Relative frequency of contacts in anterior maxillary teeth

Among the anterior maxillary teeth, canines showed the greatest relative frequency of occlusal contacts (Fig. 1). In all teeth in both groups, the relative frequency of occlusal contacts was greater with MBF. Except for the left maxillary canine in MBF, occlusal contacts were more frequent in the SDA group.

### Contact rates for anterior maxillary teeth on individual level

**Fig. 2:**
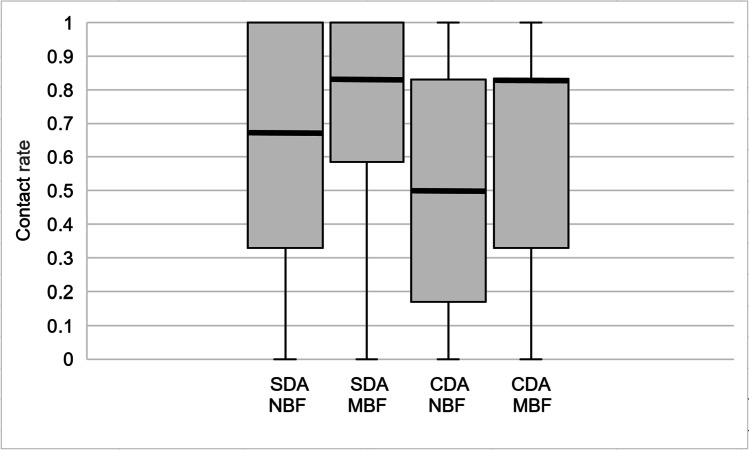
Contact rates for anterior maxillary teeth. Median, quartiles, minima, and maxima

The values were a multiple of one sixth because all participants had six anterior maxillary teeth. Tests of normality showed no normal distribution (Kolmogorov–Smirnov Test, Shapiro–Wilk Test). The range was 0 to 1. The medians for the SDA group were 0.67 (NBF) and 0.83 (MBF), for the CDA group 0.50 (NBF) and 0.83 (MBF). The interquartile ranges were large (Fig. 2). Only under NBF, the contact rates were significantly higher in the SDA group (Mann–Whitney *U* test). Within both groups, the contact rates were significantly higher in MBF (Wilcoxon Test).

### Generalized linear model for occlusal contacts of anterior teeth

A generalized linear model for a binomial response (logistic regression) was used to determine whether the occurrence of occlusal contacts on anterior maxillary teeth as the dependent variable was associated with the independent variables gender, age, and type of dental arch. For occlusal contact of an anterior maxillary tooth under NBF, gender and type of dental arch were statistically significant (Table 1). The odds ratio (OR) for female gender was estimated as 0.660. The SDA had an estimated OR of 2.277. For occlusal contact of an anterior maxillary tooth under MBF, only the type of dental arch was statistically significant. The OR for SDA was estimated as 1.691.

**Table 1:** Parameter estimates for tooth-related occlusal contacts

	Normal bite force			Maximal bite force		
Variables	*P*	Odds ratio	95% confidence interval	*P*	Odds ratio	95% confidence interval
Female	.028	.660	.456–.955	.269	.801	.541–1.187
Male		1			1	
Age	.748	.995	.963–1.028	.989	1.000	.966–1.035
Female * age	.273	.975	.931–1.020	.052	.952	.905–1.000
Male * age		1			1	
SDA	.000	2.277	1.573–3.296	.009	1.691	1.141–2.506
CDA		1			1	

## Discussion

In this case–control study, the study hypothesis of more anterior occlusal contacts in SDAs compared to CDAs was confirmed. Relative to the rate of anterior teeth in contact, this finding applies to NBF only. However, a generalized linear model showed that the odds of an anterior tooth to have an occlusal contact are greater for SDA conditions both in NBF and MBF.

The dependence of occlusal patterns on biting forces is known. For fully dentate patients, the greatest difference was found in anterior teeth when comparing biting with light and hard pressure [20, 21]. In a study with a computerized system, similar results were obtained [22]. Young adults with full dentition were asked to clench on the pressure sensitive sheets with different voluntary contractions. With increasing clenching strength, the occlusal force and the occlusal contact area in all regions increased albeit to a lesser extent in anterior teeth than in molars.

A clinical 6-year follow-up study focused on occlusal stability and the distribution of occlusal contacts with SDA and CDA [19]. Without alteration of the bite force, occlusal contacts of maxillary incisors were tested with occlusal registration strips. More anterior occlusal contacts were recorded in the SDA group, but without statistical significance. Over 6 years, no significant difference was found. In our study, the only verified time parameter was the presence of the SDA for at least 3 years. It was chosen as a safety margin to ensure a defined time span for initial adaptation after molar loss. However, there is no reliable evidence on the course and duration of adaptive changes after removing posterior teeth. It has been assumed that the adaptive changes in SDAs happen shortly after the loss of molar support [19]. Regarding the participants with SDA in our study, we presume that the adaptive processes were already widely completed. We consider the differences between SDA and CDA caused by a manifestation of advanced adaptation to function without molar support that might remain stable for a long time.

The significance of the type of dental arch in the generalized linear model was in line with the differences found relative to the contact rates in NBF. Different from the results for the contact rates, the odds for a contact of an anterior tooth in SDAs were still greater in MBF, although to a lesser extent. The analysis also revealed a gender effect. The reason for lower odds of anterior contacts in women with NBF could be that the general biting forces are lower [23]. It could be supposed that women tend to bite lighter when asked to bite normal. As they clench purposely harder, the statistical significance of gender disappeared.

In summary, we found that the differences in anterior occlusal contacts between SDA and CDA tend to even out with higher forces. However, maximum bite forces are an intentionally forced condition, spontaneously not occurring during holding and manipulating food by healthy individuals without coexisting bruxism [24]. Periodontal mechanoreceptors are responsible for adjusting the muscle tension in the chewing process. After blocking them with anesthesia, the food holding forces increased up to 3.5 times, whereas food splitting forces remained the same [25]. The splitting forces do not increase until the hardness of food rises [26]. Due to the protecting mechanisms of the periodontal tissues, most of the biting activities are performed under submaximal forces. Therefore, the results with NBF are considered more relevant.

A number of compensatory mechanisms in SDAs have been discussed, among them more interdental spacing in the premolar region, more anterior contacts [17], and more occlusal wear in premolars [16]. To what extent they contribute to a new equilibrium [17] cannot be derived from our results. However, we presume that all of them play a role in keeping up the oral functions. Individuals with SDAs might tend to utilize the anterior teeth to partially take over functions of the posterior teeth. Probably, individuals with SDA develop stronger habitual bite forces to improve chewing efficacy as they have less teeth. In an experimental in vitro and in vivo study with simulations of SDAs, the tooth related bite force in premolars and anterior teeth increased with missing molar support [18]. However, no evidence for overloading temporomandibular joints and teeth in SDAs was found. The authors conclude that the neuromuscular system is designed to control the clenching strength depending on the occlusal conditions. A clinical study examined the masticatory performance of individuals with SDA, SDA and PRDP, and CDA as control [27]. Although individuals with SDA showed lower contact areas and occlusal forces, no differences in the masticatory performance were reported. The authors presume that individuals with SDA may preserve their masticatory function by shifting chewing activities towards premolars and anterior teeth. In summary, there are a number of hints in terms of adaptive changes after the loss of molar support. Our results contribute to the knowledge in this field.

Among the weaknesses of this study is the potential bias caused by group differences concerning periodontal condition and intermaxillary relationship. The subjectiveness of perceived bite force under commands to bite with normal and maximal strength is a further limitation, as the exerted forces are not measurable. Regarding reliability and reproducibility, the studies which used EMG feedback to train their subjects to control muscle tension and bite forces can be considered superior [28]. Testing with foils involves further sources of uncertaincies. A laboratory study with 8-µm shimstock foil revealed that occlusal gaps of 6 to 8 µm may still be interpreted as contacts [29]. In addition, the removal forces applied by the examiner are not standardized. There are also advantages of foil testing such as an easy examination scarcely impairing the tested individual’s functions. A further risk of bias relates to individuals with preexisting high contact rates in anterior teeth prior to posterior tooth loss who may be more apt to accept an SDA leading to an overrepresentation in the SDA group.

## Conclusions

The findings of this study suggest effective compensatory mechanisms relative to the occlusal function in individuals with SDA supporting the SDA concept.
